# Feasibility and Effectiveness of Mini-Clinical Evaluation Exercise (Mini-CEX) in an Undergraduate Medical Program: A Study From Pakistan

**DOI:** 10.7759/cureus.29563

**Published:** 2022-09-25

**Authors:** Shameel Shafqat, Isbaah Tejani, Muhammad Ali, Hemaila Tariq, Saniya Sabzwari

**Affiliations:** 1 College of Medicine, Aga Khan University Medical College, Karachi, PAK; 2 Family Medicine, Aga Khan University Hospital, Karachi, PAK

**Keywords:** faculty-student interaction, clinical faculty, undergraduate medical education, feedback, mini-cex

## Abstract

Background

In clinical settings, direct observation (DO) with feedback is an effective method to assess and improve learner performance. One tool used for DO is the mini-clinical evaluation exercise (Mini-CEX). We conducted a study to assess the effectiveness and feasibility of Mini-CEX for medical students at Aga Khan University, Karachi.

Methods

Utilizing a purposive sampling technique, a total of 199 students in six core clerkships of Years 3 and 4 were selected for this study. Participating faculty underwent training workshops for use of Mini-CEX and feedback strategies. Each student was assessed twice by one faculty, using a modified version of the Mini-CEX, which assessed four domains of clinical skills: Data Gathering, Communication, Diagnosis/Differential, and Management Plan and Organization. Feedback was given after each encounter. Faculty and students also provided detailed feedback regarding the process of DO.

Data were analyzed using Statistical Package for Social Sciences (SPSS) version 26 (IBM Corp., Armonk, NY, USA), with categorical variables arranged as frequencies and percentages. The Chi-squared test was used for further statistical analyses, and a P-value of < 0.05 was considered statistically significant. Effectiveness was assessed via a change in student performance between the first and second Mini-CEX, and feasibility was assessed via qualitative feedback.

Results

We obtained three sets of results: Mini-CEX forms (523), from which we included a total 350 evaluations for analysis, 216 from Year 3 and 134 from Year 4, and feedback on DO: student (70) and faculty (18). Year 3 students performed significantly better in all foci of the Mini-CEX between the first and second assessment (P ≤ 0.001), whereas in Year 4, significant improvement was limited to only two domains of the Mini-CEX [Communication of History/Physical Examination (P = 0.040) and Diagnosis/Differential and Management Plan (P < 0.001)]. Students (65.7%) and faculty (94.4%) felt this exercise improved their interaction. 83.3% faculty recommended its formal implementation compared to 27.1% of students, who reported challenges in implementation of the Mini-CEX such as time constraints, logistics, the subjectivity of assessment, and varying interest by faculty.

Conclusion

Direct observation using Mini-CEX is effective in improving the clinical and diagnostic skills of medical students and strengthens student-faculty interaction. While challenges exist in its implementation, the strategic placement of Mini-CEX may enhance its utility in measuring student competency.

## Introduction

Medical education has undergone fundamental changes over the past few decades. There is a pedagogical move towards competency-based teaching/learning to achieve specific learning outcomes [1]. In clinical settings, direct observation (DO) of medical student performance is a vigorous method of formative and summative assessment, and is a major assessment tool for outcomes in a competency-based medical education [2]; for example, it has shown to provide an opportunity to gauge knowledge, skills, and clinical reasoning during patient care [3]. Feedback from this interaction influences and improves students’ performance and skills.

The Mini-Clinical Evaluation Exercise (Mini-CEX) has been used as an assessment tool to determine clinical competency by DO. It was first introduced and used to evaluate Internal Medicine Residents, as an alternative to other methods of examinations via DO [4,5]. Multiple studies have argued for the use of Mini-CEX amongst medical students as a method of assessment. The Mini-CEX was found to be a feasible assessment tool during busy clerkships such as Inpatient Psychiatry, and it helped improve students’ counseling skills [6]. In another study, students and faculty reported one-on-one interaction as the most important advantage of the Mini-CEX [7]. The Mini-CEX does have limitations: time constraints secondary to high patient volumes and a limited time for patient interaction, and health system restrictions [7,8]. Despite these challenges, the Mini-CEX has proven its potential as a cost-effective way of giving students constructive feedback in a structured manner [9].

The Aga Khan University (AKU) Karachi, a frontier institution in the country, has a five-year program for Undergraduate Medical Education (UGME). Ongoing assessment in clinical clerkships is done via a form that evaluates 13 attributes of knowledge, skills, and professionalism. Key stakeholders, i.e., students and faculty, however report that opportunities for DO with feedback are limited [3]. UGME Program reviews have also recommended strengthening opportunities for DO during patient care.

We, therefore, conducted this study to assess the effectiveness and feasibility of using Mini-CEX for the DO of learners in the UGME program at AKU. Furthermore, we observed its role in student-faculty interaction and contribution to competency-based learning.

## Materials and methods

Our study was conducted during the academic year of 2018-2019, in the UGME program of Aga Khan University in Karachi, Pakistan. Approval was obtained from the Institutional Ethics Review Committee (ERC #2018-0516-662), and an initial survey was conducted amongst 55 local students (approximately half from Years 3 and 4 each), who reported a substantial lack of DO opportunities during their clinical clerkships (Table 1).

**Table 1:** Pre-study survey about Direct Observation Opportunities.

Year-Wise Clerkships	History observed less than 50%	Examination observed less than 50%	Diagnosis observed/discussed less than 50%	Management observed/discussed less than 50%
Year 3				
Internal Medicine	13.2	55.3	52.6	65.5
Family Medicine	5.88	8.82	29.4	30.8
General Surgery	8.33	25.0	41.7	45.5
Year 4				
Pediatrics	0.00	9.09	0.00	4.55
OBGYN	11.54	53.9	15.4	34.6
Psychiatry	18.2	57.9	9.09	22.73

Using a purposive sampling technique, we selected 199 students from the early clinical Years 3 and 4, rotating through six core clerkships: Medicine, Surgery, and Family Medicine in Year 3 and Psychiatry, Pediatrics, and Obstetrics and Gynecology (OBGYN) in Year 4. Clerkships less than four weeks were excluded from the study to ensure an appropriate opportunity for conducting the Mini-CEX exercise.

All 199 students rotating in the said clerkships were oriented to the Mini-CEX pilot program at the beginning of the academic year and Mini-CEX was implemented as a formative assessment tool within existing clinical interactions, with real patients. Clerkship coordinators and department chairs were asked to identify four to six faculty members to participate in this study. These faculty members attended mandatory training workshops to standardize DO skills and feedback strategies; workshops were conducted by medical educationists who had prior experience with the Mini-CEX. We also engaged clinic administration during the planning meetings to ensure their understanding and support. We obtained written informed consent from all participating faculty members and students. We used the Mini-CEX in both inpatient and outpatient settings. In clinics, DO was completed within the first hour of the clinic, to ensure maximum opportunity for student-faculty interaction and minimal disruption of patient flow (done with the help of clinic staff). Each student had two Mini-CEXs by the same clinical faculty within one clerkship to assess its effect on student progress.

**Figure 1:**
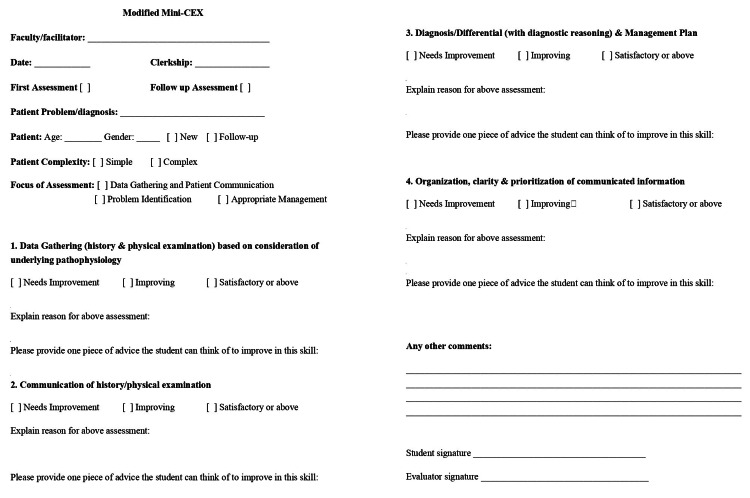
Modified Mini-CEX Form

We utilized an internally validated, modified version of the Mini-CEX for DO. Four domains of clinical skills were included: Data Gathering (history and physical examination), Communication (of history/physical examination), Diagnosis/Differential (with diagnostic reasoning), and Management Plan and Organization. These were assessed on a 3-point Likert scale (Needs Improvement, Improving and Satisfactory or Above), with justification required for each assessment and written feedback to improve that skill (Figure 1); effectiveness was assessed via a change in student performance between the first and second Mini-CEX. We also obtained feedback from students and faculty at the end of the study through specially designed and internally validated forms, and assessed the feasibility of the tool qualitatively via comments from students and faculty about ease and time of completion in real-time [10].

Analysis

Data were analyzed using Statistical Package for Social Sciences (SPSS) version 26 (IBM Corp., Armonk, NY, USA). All Mini-CEX forms were coded to maintain confidentiality. We arranged categorical variables as frequencies and percentages. The Chi-square test was used for further statistical analyses. Forms with missing data were excluded, and a P-value of <0.05 was considered statistically significant. We compiled feedback questionnaires and categorized them according to the Likert scale. Open-ended questions were reviewed, and comments were collated and summarized into two themes of ‘Strengths’ and ‘Weaknesses’ of Mini-CEX for DO, as perceived by students and faculty. 

## Results

A total of 523 Mini-CEXs were performed over the course of the year, with 306 (58.5%) from Year 3 and the remaining 217 (41.5%) from Year 4. Eighteen faculty members participated (Table 2). For our final analysis, we included a total of 350 (66%) evaluations in which both first and second assessments were obtained for the individual clinical years to identify changes in student performance, as per our methodology. Of these 216 (61.7%) were from Year 3 and 134 (38.3%) from Year 4 students, with 172 (46.1%) males and 132 (43.4%) females. In Year 3, the Family Medicine department conducted the highest DOs via Mini-CEX, with 182 (84.3%) evaluations, whereas General Surgery and General Medicine completed 18 (8.3%) and 16 (7.4%) forms, respectively. From Year 4, 68 (50.7%) evaluations were included from Psychiatry, while Pediatrics and OBGYN completed 40 (29.9%) and 26 (19.4%) assessments, respectively. The majority of students were assessed on initial patients (240; 75.4%). A total of 309 forms had the complexity of patient’s information filled: 212 (68.6%) of students were evaluated on simple patients, while 97 (31.4%) did the Mini-CEXs with complex patients. Of note, the majority of complex patients (45; 46.4%) were found in Psychiatry, followed by Family Medicine (28; 28.9%), and General Medicine (9; 9.3%).

**Table 2:** Participating faculty rank and position.

Faculty Rank Position	Total; N= 18 (n%)	Year 3; N=11 (n%)	Year 4; N= 7 (n%)
Chair	2 (11.1)	2 (18.2)	0
Associate Professor	5 (27.8)	3 (27.3)	2 (28.6)
Assistant Professor	6 (33.3)	2 (18.2)	4 (57.1)
Consultant	1 (5.6)	1 (9.1)	0
Senior Instructor	2 (11.1)	1 (9.1)	1 (14.3)
Lecturer	2 (11.1)	2 (18.2)	0

We assessed the effectiveness of the Mini-CEX individually for Years 3 and 4.

In Year 3, we found a statistically significant improvement between the first and second Mini-CEX in all foci of assessment (P ≤ 0.001)(Table 3), whereas in Year 4, there was a significant improvement in only two domains of the Mini-CEX [Communication of History/Physical Examination (P = 0.040) and Diagnosis/Differential and Management Plan (P < 0.001)] (Table 4).

**Table 3:** Comparison of student performance in first and second assessments for Year 3. *P <0.05 is considered significant.

Variables	Total; N=216 (n%)	First Assessment; N=108 (n%)	Second Assessment; N= 108 (n%)	P-Value
Focus of Assessment				
Data Gathering and Patient Communication	181 (83.8)	91 (84.3)	90 (83.3)	0.854
Problem Identification	82 (38.0)	33 (30.6)	49 (45.4)	0.025*
Appropriate Management	22 (10.2)	9 (8.3)	13 (12.0)	0.368
Data Gathering				<0.001*
Needs Improvement	59 (29.2)	48 (48.0)	11 (10.8)	
Improving	51 (25.2)	11 (11.0)	40 (39.2)	
Satisfactory or above	92 (45.5)	41 (41.0)	51 (50.0)	
Communication of History/Physical Examination				<0.001*
Needs Improvement	39 (20.7)	32 (34.0)	7 (7.4)	
Improving	44 (23.4)	13 (13.8)	31 (33.0)	
Satisfactory or above	105 (55.9)	49 (52.1)	56 (59.6)	
Diagnosis/Differential and Management Plan				<0.001*
Needs Improvement	35 (23.0)	27 (36.0)	8 (10.4)	
Improving	11 (14.7)	11 (14.7)	28 (36.4)	
Satisfactory or above	37 (49.3)	37 (49.3)	41 (53.2)	
Organization of Information				0.001*
Needs Improvement	20 (13.2)	17 (23.0)	3 (3.8)	
Improving	35 (23.0)	11 (14.9)	24 (30.8)	
Satisfactory or above	97 (63.8)	46 (62.2)	51 (65.4)	

**Table 4:** Comparison of student performance in first and second assessments for Year 4. *P <0.05 is considered significant.

Variables	Total; N=134 (n%)	First Assessment; N=67 (n%)	Second Assessment; N= 67 (n%)	P-Value
Focus of Assessment				
Data Gathering and Patient Communication	105 (78.4)	49 (73.1)	56 (83.6)	0.142
Problem Identification	92 (68.7)	43 (64.2)	49 (73.1)	0.264
Appropriate Management	53 (39.6)	23 (34.3)	30 (44.8)	0.216
Data Gathering				0.298
Needs Improvement	13 (9.9)	9 (14.1)	4 (6.0)	
Improving	33 (25.2)	15 (23.4)	18 (26.9)	
Satisfactory or above	85 (64.9)	40 (62.5)	45 (67.2)	
Communication of History/Physical Examination				0.040*
Needs Improvement	8 (6.1)	6 (9.4)	2 (3.0)	
Improving	36 (27.5)	22 (34.4)	14 (20.9)	
Satisfactory or above	87 (66.4)	36 (56.3)	51 (76.1)	
Diagnosis/Differential and Management Plan				<0.001*
Needs Improvement	9 (8.0)	9 (17.0)	0	
Improving	47 (42.0)	23 (43.4)	24 (40.7)	
Satisfactory or above	56 (50.0)	21 (39.6)	35 (59.3)	
Organization of Information				0.455
Needs Improvement	6 (5.3)	4 (7.1)	2 (3.4)	
Improving	29 (25.4)	16 (28.6)	13 (22.4)	
Satisfactory or above	79 (69.3)	36 (64.3)	43 (74.1)	

Cronbach’s Alpha was calculated for feedback forms, showing a value of 0.885 and 0.912 for the student and faculty feedback forms, respectively.

Student feedback

**Figure 2:**
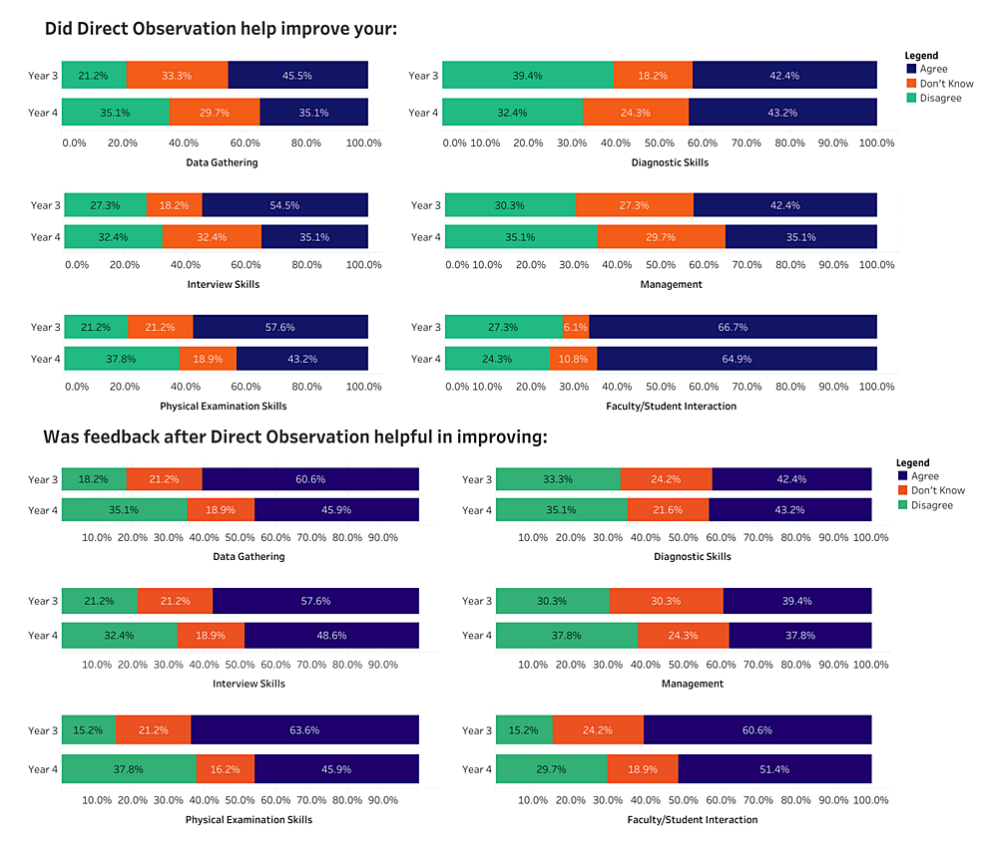
Student feedback about the implementation of mini-clinical evaluation exercise (Mini-CEX) for direct observation.

Seventy students provided feedback on the implementation of Mini-CEX as a tool for DO, with 37 (52.9%) from Year 4. Further details of student feedback are provided in Figure 2. Students based effective utilization of Mini-CEX on completion of the process within clerkships. In Year 3, 81.8% reported that Mini-CEX was most effectively utilized in Family Medicine and Pediatrics (67.6%) in Year 4. Almost half the students (48.6%) felt intimidated by the faculty observing them, with 27.1% of students recommending formal implementation of the Mini-CEX in UGME.

Faculty feedback

**Figure 3:**
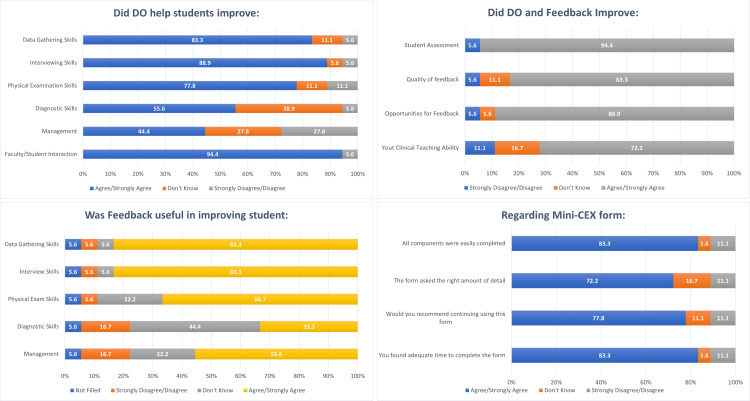
Faculty feedback about the implementation of mini-clinical evaluation exercise (Mini-CEX) for direct observation (DO).

All faculty members provided feedback (Figure 3). Almost all (94.4%) faculty reported that this exercise improved faculty/student interaction, and 83.3% formally recommended the implementation of this project across the UGME curriculum.

We combined the comments received from students and faculty, into themes of strengths and weaknesses of the Mini-CEX form (Table 5). Students' real-time feedback was a strength of Mini-CEX, while a limited time duration due to busy clinics was considered a weakness by students and faculty alike.

**Table 5:** Strengths and weaknesses identified through feedback.

Feedback	Faculty	Students
Strengths Identified		
Improved student/faculty interaction	4	4
Opportunity to observe students’ performance	6	10
Opportunity to give real-time feedback	3	32
Weaknesses identified		
Time constraints	12	16
Subjectivity	1	12
Administrative issues	3	2
Student/Faculty lack of interest	2	7
Intimidating Faculty	-	15

## Discussion

Our study assessed the effectiveness and feasibility of the Mini-CEX for direct observation of students in the undergraduate medical program at AKU. We also explored the effects of this exercise on student-faculty interaction and obtained stakeholder feedback on strengths and challenges. A recent study from the Norwegian University of Science and Technology evaluated the use of Mini-CEX in their undergraduate program, concluding with positive recommendations for the implementation of this tool for formative assessment [11]. In our region, there is a paucity of data regarding formative assessments in UGME, with only one study from 2015 [12]. Therefore, we hoped to add to the existing literature, a way forward in planning and implementing Mini-CEX in busy clinical programs.

In our experience, the implementation of this project formalized and improved opportunities for the DO of students. We found some similarities and differences from previous studies: The effectiveness of the Mini-CEX seemed to differ in both years. For example, Mini-CEX demonstrated a visible improvement across all domains of assessment for Year 3 students, whereas a significant difference in performance in Year 4 was restricted to only two foci. Norcini et al. cited contrasting results [4]. We believe this is likely due to a higher level of learners i.e., post-graduate trainees, and possibly an increased number of observations. In our understanding, both these results reflect the level of the learner and may help in planning the implementation of Mini-CEX for undergraduate learners. We advocate repeated use of Mini-CEX to allow students to work on their skills [13] and shift the focus of the Mini-CEX to its different domains over time based on the academic progression of the student throughout medical school.

Most of the completed Mini-CEXs came from the Family Medicine clerkship and least from the General Medicine clerkship. We believe that this difference is arbitrary, as another study cited the highest number of responses from General Medicine [14]. In our context, the greater complexity of patients in Medicine clinics may have limited Mini-CEX opportunities for third-year students.

The key strength of the DO observation in our study lay in the increased student-faculty interaction, making Mini-CEX a valuable tool to aid such contact in busy clinical settings [15]. As stated by a student, “Two spaced-out evaluations helped me get a better understanding of my progress in the clinical rotation”. Proximate feedback that resulted from this interaction was another strength of this project. As reported previously [16-18], formative assessments and feedback are considered effective tools for improving skills [19], therefore lending support to the implementation of DO via Mini-CEX. However, despite the value of feedback in improving learner performance, DO in undergraduate medical education remains suboptimal [20]. Institutions planning to implement DO must seek to identify individualized ways to optimize utilization.

The quality of feedback in DO also matters; learners benefit more from specific feedback than numerical scores [21]. Timely and specific feedback is often difficult in un-observed student-patient encounters. Mini-CEX provides an opportunity for consultants to give not only immediate but specific feedback to improve the clinical judgment of learners [22], as also seen in our study. As quoted by a student, “In its true sense, this gave us the chance to interact with faculty one on one. I always wanted my consultants to look at my examination technique and the way I interacted with patients so they could identify my mistakes/strong points”. Feedback from faculty is shown to trigger positive emotional reactions and self-reflection among students, encouraging them to be more motivated toward learning [23].

The formative nature of this interaction was appreciated by both students and faculty [24]. The resulting feedback allowed better case correlation and understanding and encouraged more active student participation, improved communication, and clarification of Urdu terminologies [25,26]. As one student stated, “It’s not graded, so there’s less pressure to perform and therefore we do what we usually do in the clinic and learn from our mistakes”.

All levels of faculty members participated, albeit their numbers were small. We believe offering more workshops that enhance understanding of the Mini-CEX tool might engage greater number of clinical faculty in conducting DOs [27]. Faculty members saw a clear benefit in using Mini-CEX for learning and feedback and believed it also helped improve their clinical teaching ability. Faculty support for Mini-CEX is found in prior studies, with another faculty cohort citing the experience as a self-learning exercise [1].

While the students found the interaction during DO beneficial, some found it daunting. As a student described, “Often felt uncomfortable with the consultant observing”. The sense of intimidation reported in our study is similar to previous citations where students and residents were uncomfortable during the Mini-CEX and/or feedback [4,21]. This could be because of a natural fear of criticism, the nature of the faculty, or past interactions. Surprisingly, in our study the junior students felt more comfortable with faculty observing them; whether this was also linked to specific clerkships was not explored. We believe that the intimidation factor markedly contributed to why a majority of students did not support the formal implementation of DO in the UGME curriculum. We suggest an early start and regular use of the Mini-CEX for direct observation and feedback as a way to overcome student discomfort [28].

We found time limitation as the major barrier to the implementation of Mini-CEX for DO and a constraint in its feasibility [29]. As reported “It was hard to manage to get Mini-CEX forms filled due to busy clinics and lack of time”. Other factors influencing feasibility were clinic volumes, space constraints, and placing ownership of the process largely on faculty members [3,30]. We propose that in busy clinical settings, the way to overcome these challenges is to create multiple opportunities for Mini-CEX and allow learners to self-identify opportunities to engage faculty. Faculty members formally engaged in education may also be tasked to perform a set number of Mini-CEXs per clerkship. Sessions to demonstrate the utility of the Mini-CEX and constant reinforcement may also improve its completion rate, and thus, feasibility [31]. Furthermore, to improve feasibility, more clerkships with a wider range of patient problems should be engaged, increasing the number of opportunities for Mini-CEX to gauge clinical competence.

Another weakness in this process identified by students was low enthusiasm and lack of uniform and constructive feedback by some faculty members. A student reported that, “The expectations of every consultant are very different, thus grading was very subjective”. Consultant expectations, and their internal state during the Mini-CEX, could influence the assessment and feedback, causing a lack of uniformity in evaluating student performance [32]. This necessitates ongoing faculty training.

Patient complexity was an additional challenge as highlighted by a student, “Sometimes, the patients were very complex, which didn’t help us perform or learn appropriately. Also, sometimes the patient started addressing the consultant directly, and it got hard to maintain communication with the patient”. The solution to this lies with more frequent observations, thereby increasing the opportunity for varied patient complexity.

Our study has some limitations. It was a single-institutional study, therefore external validity may be affected. Another limitation in our study was the small number of faculty engaged in the process. A self-selection bias in faculty members with an inherent interest in education may have influenced their affirmation of the DO process. We may have seen different results with a larger faculty cohort.

## Conclusions

The pandemic has had a transformative effect on health education. Medical students being brought to the frontlines of care and a broader use of their communication skills for implementation of effective telemedicine makes the adoption of a competency-based education even more compelling. DO is an important tool to assess competency and should be strategically implemented in the assessment program of undergraduate medical education. Mini-CEX is a valid tool for formative assessment of students' clinical skills and communication and should be incorporated purposefully in curricula that aim for competency attainment. However, factors such as time constraints and clinic patient influx limit its completion rate, and by extension, the feasibility of the tool itself.

Therefore, we believe that the way forward is to incorporate planned opportunities for multiple Mini-CEXs into the curriculum and ensure that faculty engaged in clinical education perform a set number of Mini-CEXs, with the executional responsibility tasked to both faculty and learners. Furthermore, we recommend early introduction of workplace-based assessments [Mini-CEX, Direct Observation of Procedural Skills (DOPS), Case-Based Discussions (CBD)] within the UGME to allow students to overcome the sense of intimidation, with identification and training of clinical faculty for DO and feedback as the best way to ensure quality assurance and optimum utilization.
